# Tuberculosis in Adolescents in Bulgaria for a Three-Year Period: 2018–2020

**DOI:** 10.3390/children9060785

**Published:** 2022-05-26

**Authors:** Natalia Gabrovska, Albena Spasova, Anabela Galacheva, Dimitar Kostadinov, Nikolay Yanev, Vladimir Milanov, Kaloyan Gabrovski, Svetlana Velizarova

**Affiliations:** 1 Department of Pediatrics, Specialized Hospital for Active Treatment of Children’s Diseases “Prof. Ivan Mitev”, Medical University–Sofia, 1606 Sofia, Bulgaria; doc_spasova@abv.bg (A.S.); filigrance@gmail.com (A.G.); sv_velizarova@abv.bg (S.V.); 2 Department of Pulmonary Diseases, Multiprofile Hospital for Active Treatment of Pulmonary Diseases “St. Sofia”, Medical University–Sofia, 1431 Sofia, Bulgaria; dimko@mail.bg (D.K.); dr.nikolay.yanev@gmail.com (N.Y.); vlmilanov@yahoo.com (V.M.); 3 Department of Neurosurgery, University Hospital “St. Ivan Rilski”, Medical University–Sofia, 1431 Sofia, Bulgaria; k_gabrovski@abv.bg

**Keywords:** tuberculosis, adolescents TB, TB epidemiology, TB diagnosis

## Abstract

Background: Each year, approximately two million adolescents and young adults in the world become infected with tuberculosis (TB). The problem is that the classification of the disease includes children in the age group 0–14 years and young adults aged 15 and over. The present study aims to analyze and compare the epidemiology and clinical presentation of TB in Bulgaria in the different age subgroups of childhood. Methods: A retrospective study was undertaken of the newly diagnosed children (*n* = 80) with TB treated onsite from January 2018 to December 2020 at the Multiprofile Hospital for Active Treatment of Pulmonary Diseases (“St. Sofia”). They were distributed into three age groups: aged 8–11 (prepuberty), aged 12–14 (younger adolescents), and aged above 15 (older adolescents). Results: A clear finding of the research indicated that adolescent children develop TB both as primary and secondary infections. In a large number of cases with the children under our care, we found enlarged intrathoracic lymph nodes as well as infiltrative changes in the lungs, i.e., we observed transitional forms. There were statistically significant differences between the age group >15 years old and each of the other two younger groups for diagnosis, the severity of intoxication, and BK spreading status. Conclusion: The course of tuberculosis in adolescence has its own specifics and differences between the three age groups in the current study.

## 1. Introduction

Following the “golden years“ in the middle of childhood, where the cases of tuberculosis (TB) decrease substantially compared to the period of early childhood, adolescence is a period of increased susceptibility to tuberculosis when both the spread of the infection with *Mycobacterium tuberculosis* and the frequency of TB tend to increase. The reasons for this trend have not been completely clarified. However, it is considered that the sex hormones, the changing social model of contact, and the immunological changes might play a role [1,2]. About 1.8 million adolescents and young people around the world develop TB each year—an issue addressed quite recently in view of the existing division of tuberculosis into “children“ (aged 0–14 years) and “adults“ (aged ≥ 15 years), disregarding the adolescents [3,4].

Puberty is one of the most dynamic stages of physiological growth, where the morphological changes in the body are most visible. Particular emphasis in TB pathogenesis during puberty is placed on the endocrine glands. Their functioning causes substantial immune deficiency in teenagers during puberty. 

The factors for compromising immunity are changes in eating habits, irregular sleep patterns, lack of physical exercise, smoking, and alcohol abuse. These factors most often affect cellular immunity. 

The basic characteristics of tuberculosis in the age of puberty are as follows:

1. Prevalence of secondary forms;
2. “Pubertal phthisis”—evidence of both primary and secondary TB;
3. Physiologically lower natural immunity;
4. The higher frequency of TB incidence among adolescents in most countries has medium and increased morbidity.

Adolescent TB patients could be identified in one of the following ways:

1. Exposure to *Mycobacterium tuberculosis* as a part of the investigation for contact tracing;
2. Manifestation of clinical symptoms or signs indicating the diagnosis of tuberculosis;
3. Presence of results positive for tuberculosis–X-ray changes or positive blood immunological tests such as Interferon Gamma Release Assay (IGRA) or Tuberculin Skin Test (TST) [5].

Most adolescents have TB of the intrathoracic lymph nodes, infiltrative TB (infiltrates, cavities, miliary tuberculosis), and pleural effusion. [6] The general symptoms include cough, fever, and weight loss. The chest X-ray findings reflect the changes in the pathogenesis occurring with age: in adolescents, the cavities and pleural effusions are more frequent, while intrathoracic lymphadenopathy or miliary disease, which are commonly observed in younger children, are met less often [7,8,9,10]. Parenchymal changes are more often apical, as in the case of adults. 

The various sociological, biological, and behavioural patterns of adolescents and young adults shape the TB epidemiology; however, little attention has been paid to understanding the severity of tuberculosis in this sub-population group. The lack of individualized data in reporting status in countries with the highest TB levels and the focus placed on the classic age limit under or above the age of 15 significantly impede the ability to understand and develop an awareness of the actual input of adolescents and young adults to the general TB morbidity [11].

In 2020, an estimated 10 million new cases of symptomatic tuberculosis were present worldwide—5.6 million men, 3.3 million women, and 1.1 million children. TB cases were found in all countries and age groups. In 2020, over a million children fell ill with TB globally. Health providers often overlook child and adolescent TB, and it can be challenging to diagnose and treat. A total of 1.5 million people died from TB in 2020 (including 214,000 people with HIV). TB is the 13th leading cause of death worldwide and the second leading infectious killer after COVID-19 (before HIV/AIDS) [3].

**Figure 1:**
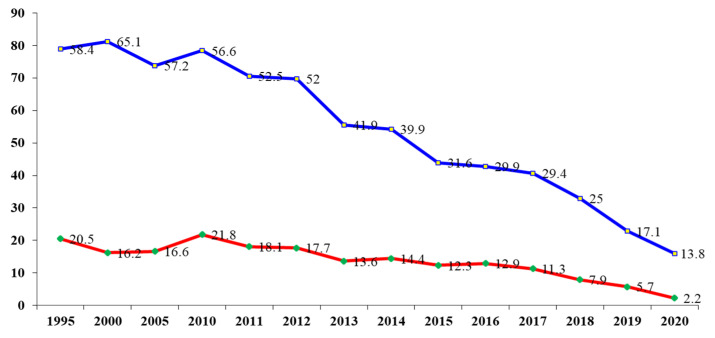
Incidence (red line) and prevalence (blue line) of tuberculosis per 100,000 children aged 0–17 in Bulgaria for 2000–2020 inclusive.

According to the latest data of the National Statistical Institute (NSI) in Bulgaria for 2020, there were 164 (13.8% per 100,000) newly diagnosed tuberculosis cases under 18 years of age—97 male and 67 female. In comparison, in 2015, they were 375 cases (31.6% per 100,000), and in 2010, 710 cases (56.6% per 100,000). There have been significant reductions in TB incidence and prevalence over the last 10 years (Figure 1) [12].

The present study aims to analyze and compare the epidemiology and clinical presentation of TB in Bulgaria in the different age subgroups of childhood.

## 2. Materials and Methods

A retrospective study was undertaken of the newly established children with tuberculosis (*n* = 80) treated onsite from January 2018 to December 2020 in the Children’s Clinic of Multiprofile Hospital for Active Treatment of Pulmonary Diseases (“St. Sofia”), Sofia, Bulgaria. The hospital is the only one in the country for the diagnosis and inpatient treatment of children with tuberculosis. All children included in the study were referred to the clinic as contacts of their relatives, patients with tuberculosis, or suspected patients due to complaints and X-ray changes. All of the hospitalized children included in the study gave a written informed consent signed by their parents. Considering there is no exposure of personal data, photographic materials etc., no special ethical approval is needed.

They were distributed, according to established age classification, into three age groups: aged 8–11 (prepuberty), aged 12–14 (younger adolescents), and aged above 15 (older adolescents) [13,14]. 

The children were studied by gender, diagnosis, presence of intoxication syndrome, evidence of contact with a TB suffering adult, presence of BCG (Bacillus Calmette–Guérin) vaccination scar (in Bulgaria, there is mandatory BCG vaccination for all newborns, and re-vaccination at the age of 7 for children with negative tuberculin sensitivity). “Microbiological confirmation” is characterized by registering and documenting positive microscopy or positive culture result. The material was taken from gastric aspirate or sputum as others described previously [15].

This research used data gathered during the routine hospital stay of the children in the clinic and the conventional set of tests to establish the presence of TB disease. Therefore, an additional requirement of informed consent beyond the standard hospital rules was unnecessary.

Comparison among the three age groups was made for sex, diagnosis, intoxication, presence of BCG scar, contact with known TB case, BK spreading status, TST status, and side and laterality of pathological findings. A chi-square or Fisher’s exact test was performed to test the distribution of several categorical variables (sex, diagnosis, presence of BCG scar, etc.). Differences between the two groups were tested by comparing the mean ranks of independent samples using Mann–Whitney U-test for the ordinal variables like intoxication. A *p*-value of <0.05 was considered statistically significant. Statistical analysis has been done by SPSS19.

## 3. Results

Eighty children in total were established in the age group 8–18 years and the subgroups, respectively: 8–11 years—34 children (52.5%), 12–14 years—29 children (36.3%) and above 15 years—17 children (21.3%). The average age of the children was 13.1 (±4.5). There were 45 boys (56.3%) and 35 girls (43.8%) in the study. 

Our results by diagnoses, presence of intoxication, presence of BCG scar, evidence of contact, presence of bacilli spreading, TST sensitivity, and X-ray changes of the site and side are shown in Table 1.

As shown in Table 1, we observed statistically significant intergroup differences in the diagnosis, degree of intoxication, and BK spreading status. All of those were significant when comparing group 3 (above 15 years old) with each of the other two younger age groups.

## 4. Discussion

Strictly speaking, our study population represents a cohort of hospitalized patients in a single institution for a three-year period. Nevertheless, the diagnosis and treatment of childhood TB in Bulgaria is highly centralized, and all diagnosed and suspected cases are referred to our department. There are no other institutions dealing with this entity for the respective time period. Moreover, there are no government or health insurance-financed centers treating TB in children outside of ours in the country, which means there are few, if any, childhood patients treated (hospitalized or outpatient) without being admitted, at least for diagnostic workup, to the department. Therefore, we considered the study population in the current study highly representative of the epidemiology of symptomatic childhood TB in Bulgaria.

In view of the small number of children, we can hardly conclude which gender prevails, but in the case of adolescents, females are reported to have a higher frequency than males, especially in countries with increased frequency of the disease, which is an exception from the general TB age profile in other age groups (higher percentage for males) [16].

It is worth mentioning that in the age group 8–11 years, TB of the intrathoracic lymph nodes prevailed in 28 children (82.4%), while the infiltrative-pneumonic TB was observed in only 2 children (5.9%). With increasing age, the TB of the intrathoracic lymph nodes shrinks at the expense of the infiltrative-pneumonic forms. With the children aged 12–14, the bronchadenitis occurred in 22 children (75.9%), and the infiltrative-pneumonic TB in 5 children (17.2%). In older adolescents (over 15) the secondary forms prevailed in 12 children (70.7%), as opposed to the bronchadenitis observed in 3 children. Adolescents and young adults have a higher risk of infection than children. Whether older adolescents have a higher risk of infection than younger ones is not explicitly clear, and it probably depends on the intensity of the transmission of the disease and its general prevalence in a certain region or age group [17,18,19]. Nevertheless, it is clear that in adolescence and younger age that TB incidence is constantly growing, and moreover, severe and late-discovered forms have been registered. 

Severe intoxication syndrome (persistent refractory fever, adynamia to prostration, greatly reduced appetite, and considerable weight loss) is more pronounced in higher age groups reaching 58.8% in adolescents aged over 15, which in turn creates obvious negligence and inattention to the symptoms of behalf of the general practitioner. The pathogenesis and the clinical presentation of TB in children, even in adolescents, are different from those in adults, with reduced sensitivity and specificity of the additional diagnostic tests, such as the microbiological testing, for example [20,21].

The examination for the presence of a BCG vaccine scar shows its absence in 44.8% to 70.6% in the highest age group, which in turn demonstrates that there is no explicit control over the BCG vaccination that has been mandatory in Bulgaria since 1951. 

Bacterial spread increases with age, with children aged 8–11 years at 8.8%, 12–14 years at 17.2%, and the group over 15 at 47.1%. The relative share of bacilli spreading is the highest in children having pulmonary forms of tuberculosis. Adolescents are often bacilli carriers, and they can transmit the disease by developing pulmonary forms. Moreover, they are able to cough and eliminate bacilli, similar to what occurs with adults [22,23]. For the period of the study, 16 (20%) children were bacilli carriers proved by microscopy or culture, which stands close to the ECDC data at 19.8% [2].

The analysis on possible contact with TB-infected adults with established contact was 73.3%, and 68.8% for its occurrence in a family. This proves once more the importance of the epidemiological survey and the early investigation of the contact individuals. 

The Mantoux tuberculin test is performed with 5 TE PPD (0.1 mL tuberculin) strictly intradermally in the upper third of the forearm. At 72 h, the transverse size of the formed infiltrate was measured. Due to the fact that BCG vaccination is mandatory in Bulgaria, the TST is negative for 0–5 mm size of the infiltrate, normal for 6–14 mm, and positive for values above 15 mm. 

In 71.4% of the examined children, we found TST above 15 mm, which shows the good diagnostic capabilities of TST. Negative TST was observed in only nine children who had very severe forms of TB, leading to immunosuppression and negative values of the TST. During treatment, these children received normal tuberculin tests. The price of the IGRA test is much higher than that of the TST as it is technically more complicated. Moreover, this includes blood draw, which is a limitation in the case of children. On the other hand, TST requires two visits in order to obtain the results within 48–72 h. International standards tend to keep TST as a standard for identifying cases of TB infection at the expense of IGRA in view of the advantages of TST in terms of cost, more reliable results with small children, and no need for laboratory resources [24].

The lower the age of the child, the more frequent the X-ray morphological changes in the lymphatic system of the lung. With increasing age, the parenchymal changes in the lungs rise accordingly, and at the age of 15 and above they reach 47.1%, approaching the X-ray changes typical for adults. In all three age groups, the X-ray morphological changes are more often bilateral or are located in the right lung, which is consistent with the literature [25]. 

Our study deals with TB patients referred to our resource center for a given period of three years. Nevertheless, according to the National Statistical Institute, the total number of patients with TB in the respective age group and period of time in Bulgaria was 109, meaning that our sample of 80 cases represented 87.2% of all TB adolescent patients in the country. In our opinion, this makes our study highly representative of the epidemiology of TB in the population level. 

In the majority of the available medical literature, the population is divided into two age groups: “children” aged 0–14 years and “adults” aged ≥15. Puberty has not been defined as a separate age group, and it has been definitely ignored in this classification. There is a need for a better understanding of the epidemiology, prevention, diagnostics, and treatment in this particular age group [1,2,26]. Of particular note is that in this age group, tuberculosis develops both as primary and secondary. In many cases with the children under our care, we found enlarged intrathoracic lymph nodes and infiltrative changes in the lungs, i.e., we observed transitional forms. 

The effective epidemiological survey and the well-organized control of the coverage of the contact individuals is a guarantee for early detection of the TB infection and active disease. 

It is also known that in the puberty period, the risk of developing active tuberculosis out of an existing latent TB infection is most serious and likely, which can be clearly perceived in the first two years after a primary infection with *Mycobacterium tuberculosis*. This period of two years is the most suitable time to initiate preventive measures as part of the treatment of latent TB infection, thus accomplishing competent prevention against the development of active TB disease in puberty age [27].

## 5. Conclusions

Our results clearly demonstrate, with statistical significance, that the course of TB differs in the age group >15 years compared to both of the other age groups. Differences between the two younger age groups, although statistically insignificant, show some specifics in the way disease develops, which in our opinion, has substantial clinical implications.

## Figures and Tables

**Table 1:** Descriptive statistics and comparison between the three age groups.

	Group I	Group II	Group III		Statistical Test	Between Group Comparison (Statistical Significance–*p*-Value)		
	8–11	12–14	Above 15	Total		I vs. II	I vs. III	II vs. III
**Diagnosis**								
TB of the intrathoracic lymph nodes	28 (82.4%)	22 (75.9%)	3 (17.6%)	53 (66.2%)	Chi-square/ Fisher’s exact test	0.526	**<0.001**	**<0.001**
Others:	6 (17.6%)	7 (24.1%)	14 (82.4%)	27 (33.8%)				
Infiltrative-pneumonic TB	2 (5.9%)	5 (17.2%)	12 (70.7%)	19 (23.8%)				
Pleuritis	3 (8.8%)	1 (3.4%)	1 (5.9%)	5 (6.3%)				
Primary TB complex	1 (2.9%)	1 (3.4%)	0	2 (2.5%)				
Extrapulmonary TB	0	0	1 (5.9%)	1 (1.3%)				
**Sex**								
Male	19 (55.9%)	16 (55.2%)	10 (58.8%)	45 (56.3%)	Chi-square/ Fisher’s exact test	0.955	0.842	0.809
Female	15 (44.1%)	13 (44.8%)	7 (41.2%)	35 (43.7%)				
**Intoxication**								
None	22 (64.7%)	19 (65.5%)	3 (17.6%)	44 (55%)	Mann–Whitney U-test	0.909	**<0.001**	**0.002**
Mild	7 (20.6%)	4 (13.8%)	4 (23.5%)	15 (18.8%)				
Severe	5 (14.7%)	6 (20.7%)	10 (58.8%)	21 (26.3%)				
**BCG scar**								
Yes	11 (32.4%)	16 (55.2%)	5 (29.4%)	32 (40%)	Chi-square/ Fisher’s exact test	0.068	0.831	0.090
No	23 (67.6%)	13 (44.8%)	12 (70.6%)	48 (60%)				
**Known contact**								
None	9 (26.5%)	5 (17.2%)	7 (41.2%)	21 (26.3%)	Chi-square/ Fisher’s exact test	0.380	0.286	0.093
Present	25 (73.5%)	24 (82.8%)						
In the family		22 (75.9%)	8 (47.1%)	55 (68.8%)				
At school	0	0	1 (5.9%)	1 (1.3%)				
Distant	0	2 (6.9%)	1 (5.9%)	3 (3.8%)				
**BK spreading**								
Negative	31 (91.2%)	24 (82.8%)	9 (52.9%)	64 (80%)	Chi-square/ Fisher’s exact test	0.453	**0.002**	**0.044**
Positive	3 (8.8%)	5 (17.2%)	8 (47.1%)	16 (20%)				
**TST**								
0–5 mm	4 (12.9%)	4 (14.2%)	3 (14.3%)	11 (13.8%)	Chi-square	0.354	0.367	0.411
6–14 mm	5 (16.2%)	3 (10.8%)	3 (14.3%)	11 (13.8%)				
>15 mm	22 (70.9%)	21 (75%)	15 (71.4%)	58 (72.5%)				
**X-ray–changes**								
Bronchadenitis	27 (79.4%)	21 (72.4%)	4 (23.5%)	52 (65%)				
Infiltrate with bronchadenitis	1 (2.9%)	1 (3.4%)	8 (47.1%)	10 (12.5%)				
Infiltrate with cavity	2 (5.9%)	4 (13.8%)	3 (17.6%)	9 (11.3%)				
Pleuritis	3 (8.8%)	2 (6.9%)	2 (11.8%)	7 (8.8%)				
Primary TB complex	1 (2.9%)	1 (3.4%)	0	2 (2.5%)				
**Location of the findings**								
Unilateral	16 (47.1%)	16 (55.2%)	9 (52.9%)	41 (51.2%)	Chi-square/ Fisher’s exact test	0.521	0.692	0.883
left	7 (20.6%)	9 (31%)	3 (17.6%)	19 (23.8%)				
right	9 (26.5%)	7 (24.1%)	6 (35.3%)	22 (27.6%)				
Bilateral	18 (52.9%)	13 (44.8%)	8 (47.1%)	39 (48.8%)				

The bold shows statistically significant results.

## Data Availability

The data presented in this study are available on request from the corresponding author. The data are not publicly available due to privacy restrictions.
